# Overexpression of transcription factor Foxa1 and target genes remediate therapeutic protein production bottlenecks in Chinese hamster ovary cells

**DOI:** 10.1002/bit.27274

**Published:** 2020-02-23

**Authors:** Audrey Berger, Valérie Le Fourn, Jacqueline Masternak, Alexandre Regamey, Iris Bodenmann, Pierre‐Alain Girod, Nicolas Mermod

**Affiliations:** ^1^ Department of Fundamental Microbiology, Institute of Biotechnology University of Lausanne Lausanne Switzerland; ^2^ Selexis SA Geneva Switzerland; ^3^ Present address: Laboratory of Microsystems LMIS4 Ecole Polytechnique Fédérale de Lausanne (EPFL) Lausanne Switzerland

**Keywords:** cell viability, CHO cells, Foxa1 transcription factor, protein folding, recombinant protein production

## Abstract

Despite extensive research conducted to increase protein production from Chinese hamster ovary (CHO) cells, cellular bottlenecks often remain, hindering high yields. In this study, a transcriptomic analysis led to the identification of 32 genes that are consistently upregulated in high producer clones and thus might mediate high productivity. Candidate genes were associated with functions such as signaling, protein folding, cytoskeleton organization, and cell survival. We focused on two engineering targets, Erp27, which binds unfolded proteins and the Erp57 disulfide isomerase in the endoplasmic reticulum, and Foxa1, a pioneering transcription factor involved in organ development. Erp27 moderate overexpression increased production of an easy‐to‐express antibody, whereas Erp27 and Erp57 co‐overexpression increased cell density, viability, and the yield of difficult‐to‐express proteins. Foxa1 overexpression increased cell density, cell viability, and easy‐ and difficult‐to‐express protein yields, whereas it decreased reactive oxygen species late in fed‐batch cultures. Foxa1 overexpression upregulated two other candidate genes that increased the production of difficult‐ and/or easy‐to‐express proteins, namely Ca3, involved in protecting cells from oxidative stress, and Tagap, involved in signaling and cytoskeleton remodeling. Overall, several genes allowing to overcome CHO cell bottlenecks were identified, including Foxa1, which mediated multiple favorable metabolic changes that improve therapeutic protein yields.

## Graphical abstract

In this work, the authors performed a transcriptomic analysis to identify genes upregulated in CHO cells producing therapeutic proteins at high levels. Among them, they identified candidate genes whose overexpression could improve the production of easy‐ and difficult‐to‐express therapeutic proteins. This included Erp27, an endoplasmic reticulum‐located protein that binds unfolded proteins and the Erp57 protein disulfide isomerase, and Foxa1, a pioneering transcription factor whose overexpression upregulated the expression of high production‐associated genes.

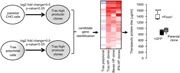

## 1 INTRODUCTION

Chinese hamster ovary (CHO) cells are currently the main host cell factory for the production of therapeutic proteins. They present several advantages, including their capacity to produce human‐like posttranslational modifications and to grow at high density in suspension in chemically defined culture media. Moreover, CHO cells are considered to be a safe host for the production of therapeutic proteins (Hansen, Pristovsek, Kildegaard, & Lee, 2017).

Although improvements in the expression of recombinant transgenes and in the bioprocessing conditions have been achieved over the past years, thus considerably enhancing productivity, cell engineering was also used to mediate increased therapeutic protein production (Farrell, McLoughlin, Milne, Marison, & Bones, 2014; Kim, Kim, & Lee, 2012; Wurm, 2004). Several studies indeed indicated that some cellular processes are not optimal in CHO cells or remain limiting for therapeutic protein production and that they may, therefore, be improved by the overexpression, downregulation, or knockout of specific genes (Baek, Kim, Park, & Lee, 2015; Hansen et al., 2017). Cell engineering has so far mainly focused on improving the time integral of viable cell concentration, by increasing the maximum viable cell density (VCD) and extending culture duration, as well as on increasing the specific productivity of CHO cells, as both parameters are determinant for the volumetric productivity of recombinant therapeutic proteins (Farrell et al., 2014; Kim et al., 2012). This was notably achieved by modulating the expression of genes involved in various cellular functions such as apoptosis, metabolism, cell cycle, and secretion (Fischer, Handrick, & Otte, 2015).

Protein folding in the endoplasmic reticulum (ER) is notably a critical step for therapeutic protein production, and it has therefore been widely investigated (Hansen et al., 2017). The protein disulfide isomerase (PDI) is an enzyme that catalyzes native disulfide bond formation, thus promoting protein folding. PDI is also involved in the rearrangement of erroneously formed disulfide bonds (Wang, Wang, & Wang, 2015). Whereas some studies reported an increase in the specific productivity of several therapeutic proteins upon PDI overexpression, other studies observed no influence or even a decrease in specific productivity or protein titer (Borth, Mattanovich, Kunert, & Katinger, 2005; Davis, Schooley, Rasmussen, Thomas, & Reddy, 2000; Hayes, Smales, & Klappa, 2010; Johari, Estes, Alves, Sinacore, & James, 2015; Mohan, Park, Chung, & Lee, 2007; Pybus et al., 2014). Another member of the PDI family, Erp57 (Pdia3), was also investigated for its potential in improving therapeutic protein production. Erp57 triggers disulfide bond formation of glycosylated proteins via interaction with the two ER lectin chaperones calreticulin (CRT) and calnexin (CNX) (Tannous, Pisoni, Hebert, & Molinari, 2015). The upregulation of CHO cell‐derived Erp57 or of both CNX and CRT was found to increase thrombopoietin specific productivity in CHO cells (Chung, Lim, Hong, Hwang, & Lee, 2004; Hwang, Chung, & Lee, 2003). However, the expression of the mouse version of Erp57 decreased the specific productivity of the α_1_‐antitrypsin and of the C1 esterase inhibitor (Hansen et al., 2015). These contradictory effects might result from distinct enzyme expression levels, origin, as well as on the expressed therapeutic protein (Hansen et al., 2017).

Given the plethora of genes whose expression may be modulated to possibly improve therapeutic protein production, more global engineering strategies have focused on the expression of transcription factors that can act as master regulators of gene expression (Gutierrez‐Gonzalez et al., 2019). Notably, overexpression of the ER stress‐related transcription factors sXBP1, sATF6, ATF4, and CHOP, successfully increased the specific productivity and/or titer of various therapeutic proteins (Becker, Florin, Pfizenmaier, & Kaufmann, 2008; Cain et al., 2013; Gulis, Simi, de Toledo, Maranhao, & Brigido, 2014; Haredy et al., 2013; Ku, Ng, Yap, & Chao, 2008; Nishimiya, Mano, Miyadai, Yoshida, & Takahashi, 2013; Ohya et al., 2008; Pybus et al., 2014; Tigges & Fussenegger, 2006); however, contradictory results were obtained upon sXBPI overexpression (Ku et al., 2008; Rahimpour et al., 2013). Furthermore, overexpression of YY1, a zinc finger transcription factor with pleiotropic effects on many cellular processes, led to an increase in antibody titer in CHO cells (Tastanova et al., 2016).

Nevertheless, despite this progress, it has been so far difficult to identify CHO cell activities whose up or downregulation may consistently yield favorable effects, irrespective of the therapeutic protein. Moreover, few of the Chinese hamster genes have been investigated for their potential in improving therapeutic protein production, indicating that opportunities remain for further developing engineered CHO cell lines. This is notably important, considering the growing number of chimerical or engineered therapeutic proteins that remain difficult to express at sufficient titers for clinical and therapeutic use (Hansen et al., 2017).

In this study, we sought to identify novel putative cell engineering targets to improve therapeutic protein production. By performing a transcriptomic analysis, we identified genes that are consistently upregulated in high producer clones and thus might be associated to high productivity, identifying candidate genes involved predominantly in signaling, cell adhesion, protein folding, cell survival, cell growth, and vesicular trafficking functions. We thus focused on Erp27, a protein that selectively binds to unfolded proteins and interacts with the disulfide isomerase Erp57 in the ER (Alanen et al., 2006; Kober et al., 2013), on Foxa1, a pioneering transcription factor involved in the development of several organs (Zaret & Carroll, 2011), and on some of Foxa1 target genes. Here we report that the expression of specific combinations of these engineering target genes yields increased cell density, viability, and specific productivity in fed‐batch cultures, resulting in higher production of easy‐to‐express as well as difficult‐to‐express therapeutic proteins and decreased reactive oxygen species (ROS), providing novel avenues towards highly efficient therapeutic protein production.

## 2 MATERIALS AND METHODS

### 2.1 Plasmids

To obtain candidate gene coding sequences (CDS), total RNA was isolated from CHO‐M cells (SURE CHO‐M Cell Line™; Selexis SA, Switzerland) using the NucleoSpin™ RNA kit (Macherey‐Nagel). Reverse transcription was performed using the GoScript Reverse transcription System (Promega). Candidate gene CDS were inserted into the pBSK_ITR_BT+_X29_ITR (pBSK_ITR) or the pBSK_ITR_Blast vectors (Le Fourn, Girod, Buceta, Regamey, & Mermod, 2014). The expression cassette is flanked by the inverted terminal sequences of the piggyBac transposon. In the pBSK_ITR_Blast vector, a blasticidin resistance gene under the control of the SV40 promoter was inserted after the hMAR X‐29. In experiments where Erp27 and Erp57 were overexpressed in difficult‐to‐express protein‐expressing cells or upon titration of Erp27 or Ca3 overexpression, the pBSK_ITR plasmid was used and cells were cotransfected with a plasmid carrying the blasticidin resistance under the control of the SV40 promoter. In other experiments, the pBSK_ITR_Blast vector was used. In experiments where Foxa1 was overexpressed, the CMV/EF1alpha promoter was replaced by a minimal cytomegalovirus (CMV) promoter for both Foxa1 and GFP expressions. The piggyBac transposase expression vector (pCS2+U5V5PBU3) was previously described (Ley et al., 2013).

### 2.2 CHO cell line development

Selexis CHO‐K1‐derived host cell line (CHO‐M cells) was adapted to grow in suspension in the chemically defined BalanCD Growth A culture medium (Irvine Scientific), and expression vectors for trastuzumab (Tras), infliximab, etanercept, and bevacizumab recombinant proteins used to transfect CHO cells were similar to that described previously (Le Fourn et al., 2014). Following transfection, puromycin selection was applied to generate corresponding stable polyclonal cell pools. To establish clonal cell lines, cell suspensions were then plated into semisolid media (CloneMedia©; Molecular Devices) and plates were incubated at 37°C and 5% CO_2_ in a humidified incubator. Growing colonies were picked using ClonePix™ FL Imager (Molecular Devices) and transferred to 96‐well plates, and then expanded in 24‐well plates and 50 ml Tubespin bioreactor (TPP, Switzerland). At each step, supernatants were analyzed for therapeutic protein production using sandwich enzyme‐linked immunosorbent assay (ELISA). Clones producing recombinant proteins at the highest levels were evaluated for growth and production performances in 10 days fed‐batch 20‐ml cultures to select the most favorable clones according to cell productivity, cell‐line stability, and product quality attributes. Clones producing the IFN‐b1a following vitamin B5 selection were described previously (Pourcel, Buron, Garcia, et al., 2020).

### 2.3 Overexpression of candidate genes in clonal cell lines

Immunoglobulin G (IgG)‐expressing CHO cell clones were obtained by cell sorting of the trastuzumab (Tras) and infliximab stable cell pools on FACSAria II (BD Biosciences), expanded and analyzed for IgG production levels by sandwich ELISA. Stable polyclonal cell pools overexpressing candidate genes were obtained by retransfecting Tras or infliximab‐producing clones with pBSK_ITR_CDS, pBlast, and pCS2+U5V5PBU3 or with pBSK_ITR_Blast_CDS and pCS2+U5V5PBU3, where CDS indicates the coding sequence of Foxa1, Tagap, Erp27, Erp57, Ca3, or Rassf9, using electroporation following the manufacturer's protocol (Neon^TM^ transfection system 100 μl Kit; Invitrogen^TM^). Cells with stable insertions were selected using 3 or 7.5 µg/ml of blasticidin (InvivoGen). For etanercept‐producing clones, single subclones co‐overexpressing Erp27 and Erp57 were isolated on semisolid media, and their production was assessed using ClonePix™ FL Imager (Molecular Devices). The cell colonies showing the widest etanercept secretion halo were isolated from the Erp27 and Erp57 overexpressing or control cell populations. Cells were maintained in suspension culture in SFM4CHO Hyclone serum‐free medium (GE Healthcare) supplemented with 5% HyClone Cell Boost 5 supplement (GE Healthcare), 8 mM l‐glutamine (PAA, Austria), and 1X HT supplement (Gibco) at 37°C in a humidified incubator with 5% CO_2_. Fed‐batch cultures and two‐tailed *t*‐test statistical evaluation of the results were performed as detailed in the Supporting Information Materials and Methods. The specific productivity was determined as the final titer divided by the integral of viable cell density (IVCD) over the culture duration.

### 2.4 RNA‐seq analysis

RNA‐seq analysis of the B5‐selected interferon β high producer clones, of the puromycin‐selected Tras polyclonal cell populations and of the parental CHO‐M cells were as described by Pourcel, Buron, Arib, et al. (2020) in the accompanying paper. These cells and the high producer clones expressing the Tras or the bevacizumab antibodies were grown for 4 days in spin tubes in batch culture. Total RNA was isolated using the NucleoSpin RNA kit (Macherey‐Nagel). The RNA quality was evaluated using the Fragment Analyzer (Advanced Analytical). RNA‐seq libraries were prepared using 0.5–1 µg of total RNA converted to complementary DNA using the Illumina TruSeq stranded messenger RNA (mRNA)‐seq reagents (Illumina). The RNA‐seq library 100 nucleotides paired‐end was sequenced on the Illumina HiSeq 2500. Reads were mapped to the CHO‐K1 transcriptome (RefSeq, 2014).

### 2.5 Reactive oxygen species analysis

The intracellular ROS level was detected by using 6‐carboxy‐2′,7′‐dichlorodihydrofluorescein diacetate (carboxy‐H_2_DCFDA; Thermo Fisher Scientific). At different days of the fed‐batch cultures, 2 million cells were incubated in phosphate‐buffered saline (PBS) containing 50 μM carboxy‐H_2_DCFDA for 30 min. Cells were then centrifuged, resuspended in 1 ml PBS, and stained with 4′,6‐diamidino‐2‐phenylindole (DAPI) to exclude dead cells. Carboxy‐H_2_DCFDA fluorescence was analyzed by flow cytometry in the DAPI‐negative cell populations (Gallios, Beckman Coulter).

## 3 RESULTS

### 3.1 Identification of genes associated with high productivity for therapeutic proteins

**Figure 1:**
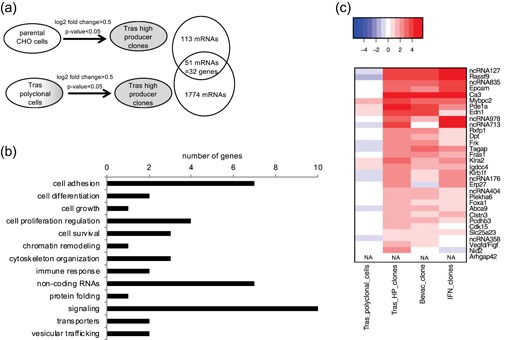
Transcriptomic analysis of genes associated with CHO cell high productivity. (a) Identification of upregulated transcripts in trastuzumab (Tras) high producer clones when compared to parental CHO cells and to the Tras polyclonal cell pool using RNA‐seq. Fifty‐one mRNAs, corresponding to 32 genes, were found to be upregulated, relative to the two control cell populations, with a log2 fold change of >0.5 and a *p* value less than .05. Three biological replicates were used for the Tras high producer clones, whereas three technical replicates (i.e., independent cultures) were used for parental CHO cells and for the Tras polyclonal cell pool. (b) Ontological analysis of the functional classes of the 32 genes upregulated in Tras high producer clones. Functions of noncoding RNAs (ncRNAs) are unknown. (c) Heatmap showing the expression profiles of the candidate genes identified as depicted in Panel a. Expression levels of the candidate genes were also assessed in cells producing at a high level the easy‐to‐express bevacizumab antibody (*n* = 1) or the difficult‐to‐express interferon β therapeutic protein (*n* = 2). Expression levels are shown as the natural logarithm of the fold change, relative to the parental CHO cells. ncRNAs were named according to Table 1. Arhgap42 expression was only detected in the Tras high producer clones. CHO, Chinese hamster ovary; mRNA, messenger RNA

The aim of this study was to identify genes displaying expression alterations that are associated with the production of therapeutic proteins at high levels by CHO cells and to test them as new cell engineering candidates for improving therapeutic protein production. For this purpose, we first carried out a transcriptomic analysis to compare three different types of cells. We analyzed CHO cell clones producing the easy‐to‐express Tras antibody at high levels while maintaining a high‐cell density, displaying an average specific productivity of 19.3 pg of Tras‐secreted per cell and per day (pg·cell^−1^·day^−1^) and an average maximum VCD of 43.3 million cells per ml. These cell lines were compared to a Tras polyclonal cell population obtained after antibiotic selection of cells stably expressing the transgenes (specific productivity of 7.4 pg·cell^−1^·day^−1^, maximum VCD of 36.3 million cells per ml), and to the parental untransfected CHO cells (Figure 1a).

Candidate genes were selected according to two criteria. We hypothesized that bottleneck CHO cell genes limiting efficient protein production would be expressed at higher levels in high producer clones than in the parental untransfected cell line. We, therefore, selected 113 mRNAs that were significantly upregulated in Tras high producer clones when compared to the parental CHO cells (Figure 1a). We also selected 1,774 mRNAs that were upregulated in the high producer clones when compared to the polyclonal Tras‐expressing cell pool. To narrow down the set of candidate genes, we then focused our analysis to the 51 mRNAs found to match both criteria, yielding 32 genes whose upregulated expression correlated to Tras high productivity (Figure 1a; Table 1). Changes in the mRNA levels of the candidate genes were further confirmed in the different samples using quantitative reverse transcription‐polymerase chain reaction (Figure S1a–d and data not shown). Surprisingly, an ontology analysis indicated that candidate protein‐coding genes were mostly associated with signaling and cell adhesion (Table 1; Figure 1b). However, we also identified genes involved in protein folding (Erp27), cell survival (Ca3, Cdk15, and Vegfd), cell growth (Clstn3), vesicular trafficking (Rassf9 and Clstn3), and cytoskeleton organization (Mybpc2, Tagap, and Arhgap42), which are cellular functions that were previously proposed to influence therapeutic protein production (Table 1; Figure 1b; Baek et al., 2015; Fischer et al., 2015; Hansen et al., 2017). Interestingly, most of these candidate genes were also upregulated in CHO cell clones producing at high level another easy‐to‐express antibody, bevacizumab, and the difficult‐to‐express interferon β protein, when compared to their expression in the parental CHO cells (Figure 1c). This indicated that candidate gene upregulation is not solely linked to cells displaying a high Tras antibody productivity, and it thus suggested that such candidate genes could be involved in high‐level production of various easy‐to‐express and difficult‐to‐express therapeutic proteins.

**Table 1:** Genes upregulated in Tras high producer clones (HPC) versus parental CHO cells and versus Tras polyclonal cells (PC)

Gene symbol^1^	Detail	Fold change (HPC/CHO cells)	Fold change (HPC/PC)	Functional classes	Foxa1 target gene^2^
Abca9	ATP‐binding cassette, subfamily A, member 9	3.45	5.08	Transporters	
Slc25a23	Solute carrier family 25 (mitochondrial carrier; phosphate carrier), member 23	2.54	2.00	Transporters	√
Erp27	Endoplasmic reticulum protein 27	4.20	3.65	Protein folding	
Ca3	Carbonic anhydrase III, muscle specific	19.86	15.68	Cell survival	√
Cdk15	Cyclin‐dependent kinase 15	2.93	2.54	Cell survival	
Vegfd	c‐Fos‐induced growth factor (vascular endothelial growth factor D)	4.35	3.90	Cell proliferation regulation/cell survival/signaling/cell differentiation	
Frk	Fyn‐related kinase	5.40	10.91	Cell proliferation regulation/signaling/cell differentiation	√
Foxa1	Forkhead box A1	2.95	3.03	Chromatin remodeling	√
Pde1a	Phosphodiesterase 1A, calmodulin‐dependent	2.79	4.93	Signaling	√
Plekha6	Pleckstrin homology domain containing family A member 6	3.51	3.56	Signaling	
Rxfp1	Relaxin/insulin‐like family peptide receptor 1	5.78	5.18	Signaling	
Edn1	Endothelin 1	17.91	10.27	Signaling	√
Tagap	T‐cell activation RhoGTPase‐activating protein	4.94	8.42	Signaling/cytoskeleton organization	
Arhgap42	Rho GTPase‐activating protein 42	12.00	10.79	Signaling/cytoskeleton organization	√
Rassf9	Ras association (RalGDS/AF‐6) domain family (N‐terminal) member 9	12.22	14.76	Vesicular trafficking	√
Clstn3	Calsyntenin 3	3.45	2.69	Cell adhesion/vesicular trafficking/cell growth	
Dpt	Dermatopontin	5.01	5.17	Cell adhesion/cell proliferation regulation	
Epcam	Epithelial cell adhesion molecule	4.56	5.56	Cell adhesion/signaling/cell proliferation regulation	√
Fras1	Fraser syndrome 1	5.99	5.28	Cell adhesion/signaling	√
Igdcc4	Immunoglobulin superfamily, DCC subclass, member 4	7.11	2.53	N/A	
Mybpc2	Myosin‐binding protein C, fast‐type (LOC100774229)	29.47	3.32	Cytoskeleton organization/cell adhesion	
Nid2	Nidogen 2 (osteonidogen)	5.88	4.37	Cell adhesion	
Pcdhb3	Protocadherin beta 3	2.83	2.43	Cell adhesion	√
Klra2	Killer cell lectin‐like receptor 2 (LOC100762405)	8.13	1.86	Immune response	
Klrb1f	Killer cell lectin‐like receptor subfamily B member 1F (LOC100757275)	4.56	6.67	Immune response	
NaN	Uncharacterized LOC103159978 (LOC103159978), hereafter called ncRNA978	5.51	5.20	Noncoding RNAs	
NaN	Uncharacterized LOC103162358 (LOC103162358), ncRNA358	2.36	2.53	Noncoding RNAs	
NaN	Uncharacterized LOC103160835 (LOC103160835), ncRNA835	10.23	6.75	Noncoding RNAs	
NaN	Uncharacterized LOC103159713 (LOC103159713), ncRNA713	3.45	4.90	Noncoding RNAs	
NaN	Uncharacterized LOC103164404 (LOC103164404), ncRNA404	2.83	2.47	Noncoding RNAs	
NaN	Uncharacterized LOC103163127 (LOC103163127), ncRNA127	4.53	5.03	Noncoding RNAs	
NaN	Uncharacterized LOC103159176 (LOC103159176), ncRNA176	5.09	9.06	Noncoding RNAs	

Abbreviations: ChIP‐seq, chromatin immunoprecipitation‐sequencing; CHO, Chinese hamster ovary; N/A, not applicable; Tras, trastuzumab.

^1^ Genes upregulated in Tras high producer cell clones versus parental CHO cells and versus Tras polyclonal cells were selected according to the following criteria: A log2 fold change greater than 0.5 and a *p* value less than .05 using the DESeq2 package (Love, Huber, & Anders, 2014).

^2^ Genes listed as Foxa1 target genes according to ChIP‐seq datasets (ENCODE Transcription Factor Targets dataset) and to low‐ or high‐throughput transcription factor functional studies (TRANSFAC Curated Transcription Factor Targets dataset) obtained using the Harmonizome web portal (Rouillard et al., 2016).

### 3.2 Erp27 overexpression alone or with Erp57 improves therapeutic protein production

We first focused on Erp27, a protein localized in the ER that selectively binds unfolded proteins (Alanen et al., 2006; Kober et al., 2013). Erp27 contains the noncatalytic b and b′ domains of PDI, but it lacks the CXXC active site required to catalyze dithiol–disulfide exchange (Alanen et al., 2006). However, Erp27 was shown to bind in vitro and in vivo to the disulfide isomerase Erp57 (Alanen et al., 2006). We, therefore, hypothesized that the Erp27–Erp57 complex might participate in therapeutic protein folding, and we sought to assess whether their expression might remain limiting for recombinant protein production even from high producer clones.

**Figure 2:**
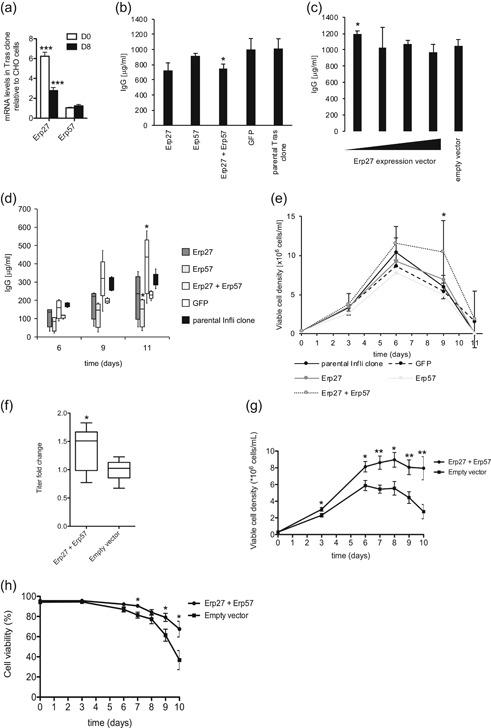
Effect of Erp27 and/or Erp57 overexpression on the production of therapeutic proteins. Clones producing easy‐ or difficult‐to‐express therapeutic proteins were stably transfected with Erp27 or Erp57 expression vectors, or cotransfected with both Erp27 and Erp57 expression vectors. Gene expression, cell growth, cell viability, and protein production were evaluated in fed‐batch cultures in stable polyclonal populations (Panels b–e) or in clones (Panels f–h) of the Erp27‐ and/or Erp57‐overexpressing cells or of the control cells. (a) Quantification of Erp27 and Erp57 mRNA levels in the parental trastuzumab (Tras) clone, represented as fold change relative to their levels in the nontransfected parental CHO cells at Days 0 and 8 of fed‐batch cultures, as assessed by qRT‐PCR. Error bars are shown as *SD*, *n* = 3. (b) The parental Tras clone was stably transfected with the Erp27 and/or Erp57 expression vectors, and the titers of the secreted Tras antibody were determined from cell culture supernatants at the end of fed‐batch cultures. Cells transfected with a GFP expression vector were used as control. Error bars are shown as *SD*, *n* = 3. (c) The parental Tras clone was stably transfected with decreasing amounts of the Erp27 expression vector (1,800, 600, 200, and 66 ng) together with an empty vector to keep the total amount of plasmid constant. Cells transfected with an empty vector plasmid were used as control. Tras titers were determined at the end of fed‐batch cultures. Error bars are shown as *SD*, *n* = 3. (d) An infliximab producer clone was characterized in terms of the secreted monoclonal antibody titers obtained during fed‐batch cultures using either the parental clone or derived cell populations obtained after transfection with the Erp27 and/or Erp57, or with the GFP expression vector, as indicated. Titers are illustrated as Tukey box‐and‐whisker diagram with median values (middle bar) and 25–50% and 50–75% quartiles (box). Whiskers extend to the lowest and highest values still within the 1.5‐fold interquartile range. (e) Viable cell density of the fed‐batch cultures analyzed in Panel d. Error bars are shown as *SD*. n ≥ 4 for Panels d and e. (f) An etanercept producer clone was stably transfected with the Erp27 and Erp57 expression vectors, or with an empty vector as control. Cell colonies were isolated using a ClonePix device, and the clones with the highest etanercept secretion halos were isolated and characterized for the etanercept titer at the end of fed‐batch cultures. The titer fold change relative to control cells is illustrated as Tukey box‐and‐whisker diagram as for Panel d. (g and h) Viable cell density and cell viability of the fed‐batch cultures analyzed in Panel f. The error bars represent the *SEM*. n ≥ 8 for Panels f–h. mRNA, messenger RNA; qRT‐PCR, quantitative reverse transcription‐polymerase chain reaction; *SD*, standard deviation; *SEM*, standard error of mean

This hypothesis was evaluated by assessing the effect of Erp27 and Erp57 overexpression on Tras secretion levels. For this purpose, clones were isolated from the Tras polyclonal population previously used for the transcriptomic analysis, and we selected the clone displaying the highest specific productivity (1.8‐fold that of the polyclonal population) while maintaining a fast cell division rate in fed‐batch cultures. Notably, the Erp27 mRNA levels of this clone were found to be upregulated by three‐ to sixfold when compared to those of parental CHO cells at Days 0 or 8 of fed‐batch cultures (Figure 2a). In contrast, Erp57 mRNA levels were similar in the CHO parental cells and Tras‐producing clone at Days 0 and 8. This clone, hereafter referred to as the parental Tras clone, was stably transfected with the Erp27 and/or Erp57 expression vectors, or with a GFP expression vector as control, and the levels of secreted Tras were evaluated during fed‐batch cultures of the polyclonal populations.

Growth and cell viability were not affected by Erp27 overexpression, whereas cell viability was decreased at Day 10 upon Erp57 overexpression (Figure S2a,b). Overall, the Tras titer levels of Erp57‐overexpressing cells and Erp27‐overexpressing cells were similar to those of control cells (Figure 2b). In contrast, whereas VCD was increased at Day 6 of the fed‐batch culture upon Erp27 and Erp57 co‐overexpression (Figure S2a), a decrease in Tras titer levels was observed (Figure 2b). We noticed that Erp27 overexpression led to a very substantial increase of the Erp27 mRNA levels when compared to the Tras‐producing clone (Figure S2c,d), suggesting that such overexpression levels may result in metabolic unbalance of this and interacting proteins, which might explain the rather reduced Tras expression. We, therefore, titrated down the amount of Erp27 expression vector used to establish stably transfected cells. Indeed, a 14% increase in the Tras levels was observed upon Erp27 reduced overexpression (Figures 2c and S2e). Overall, we concluded that Erp27 moderate overexpression increased Tras production.

As the transcriptomic analysis indicated that Erp27 mRNA expression was also increased in clones expressing the difficult‐to‐express interferon β at a high level (Figure 1c), we further assessed the effects of Erp27 and Erp57 overexpression on the production of difficult‐to‐express therapeutic proteins. The infliximab chimerical immunoglobulin (Infli) and the etanercept Fc‐fusion were used as two additional examples of difficult‐to‐express therapeutic proteins. The Infli titers were unaffected upon Erp27 overexpression and rather reduced at Day 11 upon Erp57 overexpression in an infliximab expressing clone (Figures 2d and S2f,g). However, co‐overexpression of Erp27 and Erp57 resulted in a 72% increase in Infli titers, relative to GFP‐expressing control cells at Day 11 of the fed‐batch cultures (Figure 2d). Moreover, Erp27 and Erp57 co‐overexpression yielded an increased VCD and cell viability at Day 9 of the fed‐batch (Figures 2e and S2h).

We also assessed the effect of Erp27 and Erp57 co‐overexpression in an etanercept‐producing clone. Single subclones coexpressing Erp27 and Erp57 were isolated and their production was assessed using a ClonePix cell colony imaging device. The cell colonies showing the widest etanercept secretion halo were isolated from the Erp27 and Erp57 overexpressing or control cell populations, and the derived cell clones were assessed for etanercept production in fed‐batch cultures. The VCD and cell viability were enhanced upon Erp27 and Erp57 overexpression, together with an extended plateau phase of the viable cells and a 37% increase of the titer (Figure 2f–h). Taken together, these results indicated that Erp27 moderate overexpression could increase the production of an easy‐to‐express therapeutic protein and that Erp27 and Erp57 combined overexpression could enhance VCD, cell viability, and the titer of cells producing different difficult‐to‐express therapeutic proteins.

### 3.3 Foxa1 overexpression increases Tras production and reduces oxidative stress

We next investigated another candidate gene, the Foxa1 transcription factor, for its potential role in increasing therapeutic protein production. Foxa1 is a pioneering regulatory protein that can bind to the compacted heterochromatin structures of silent genes, where it can initiate chromatin remodeling steps that lead to gene expression (for a review, see Zaret & Carroll, 2011). It is involved in the development of different organs such as the liver, pancreas, lungs, and prostate (Friedman & Kaestner, 2006). Thus, we hypothesized that Foxa1 might activate a transcriptional program favorable for the production of therapeutic proteins such as Tras.

**Figure 3:**
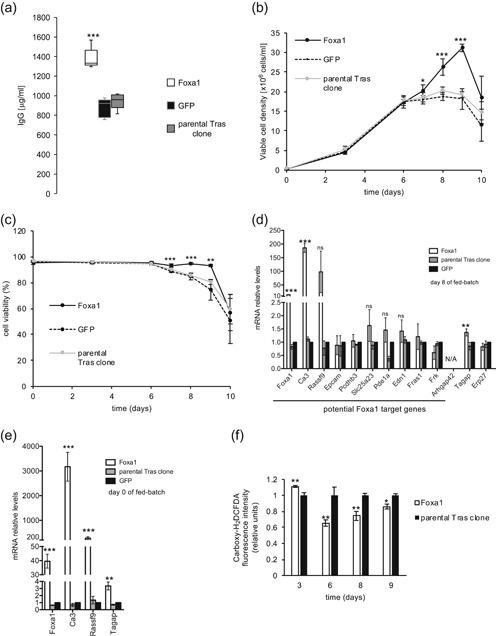
Effect of Foxa1 overexpression on trastuzumab (Tras) production. The parental Tras clone was stably transfected with the Foxa1 or GFP expression vector. (a) The Tras titers of the resulting polyclonal populations were determined after 10 days of fed‐batch cultures. Viable cell density (b) and cell viability (c) were evaluated throughout fed‐batch cultures. *n* = 5 for Panels a–c. Titers are illustrated as a Tukey box‐and‐whisker diagram as described for Figure 2, whereas error bars are shown as *SD* (Panels b and c). (d) An RT‐qPCR analysis of the mRNA levels of Foxa1 target genes and other relevant genes identified in Figure 1 was performed on Foxa1‐overexpressing cells, GFP‐expressing cells, or the parental Tras clone at Day 8 of the fed‐batch culture. Error bars are shown as *SD*, *n* = 3. Arhgap42 expression could not be detected in these samples. (e) RT‐qPCR quantification of Foxa1, Ca3, Rassf9, and Tagap mRNA levels in Foxa1‐overexpressing cells, GFP‐expressing cells, or in the parental Tras clone at Day 0 of the fed‐batch. Error bars are shown as *SD*, *n* = 3. (f) Evaluation of intracellular ROS levels using carboxy‐H_2_DCFDA in Foxa1‐overexpressing cells and in parental Tras clone at Days 0, 3, 6, 8, and 9 of the fed‐batch cultures. Error bars are shown as *SD*, *n* = 3. mRNA, messenger RNA; qRT‐PCR, quantitative reverse transcription‐polymerase chain reaction; ROS, reactive oxygen species; *SD*, standard deviation

Consistently, Foxa1 mRNA expression was increased in the Tras clone compared to the parental CHO cells at Days 0 and 8 of fed‐batch cultures, with an upregulation of 1.5‐ and 2.1‐fold, respectively (Figure S3a). A threefold upregulation was observed in the Tras high producer clones, relative to the parental CHO cell controls in the transcriptomic analysis, thus indicating that Foxa1 expression may be further increased (see Table 1). We, therefore, stably transfected the Tras‐producing clone with a Foxa1 expression vector. The stable expression of Foxa1 under the control of the strong CMV/EF1alpha promoter resulted in elevated cell death during the antibiotic‐mediated selection (data not shown). However, the substitution of this strong promoter by a minimal CMV promoter abrogated this unwanted effect, and a 57% increase in the final Tras titer was obtained upon Foxa1 overexpression (Figure 3a), whereas Foxa1 mRNA was upregulated by 40‐ and 14‐fold at Days 0 and 8 of fed‐batch cultures, respectively (Figure 3d,e). Although cell growth in fed‐batch cultures was similar when comparing Foxa1‐overexpressing to control cells up to Day 6, Foxa1‐overexpressing cells continued to divide up to Day 9, reaching an average VCD of 31 million cells per ml, and control cells peaked at 19 million cells per ml at Day 8 (Figure 3b). Moreover, the viability of Foxa1‐overexpressing cells remained above 90% until Day 9, whereas the control cell viability decreased from Day 7 and was below 75% at Day 9 (Figure 3c). Notably, a 28% increase in the specific productivity was observed upon Foxa1 overexpression, with specific productivity of 11 pg·cell^−1^·day^−1^ versus the specific productivity of 8.6 pg·cell^−1^·day^−1^ for the GFP control cells (*p* = .008). This indicated that the Foxa1‐mediated increase in titer was due to both an increased time integral of the VCD and to increased specific productivity.

### 3.4 Ca3, Rassf9, and Tagap are upregulated upon Foxa1 overexpression in Tras‐producing clone

The fact that Foxa1 is a pioneering transcription factor suggested that it might directly increase the transcription of Tras heavy chain (HC) and light chain (LC) transgenes. However, we did not observe any significant change in the Tras HC and LC mRNA levels upon Foxa1 overexpression (Figure S3b). We, therefore, hypothesized that the Foxa1‐mediated increase of Tras titer may result from the transcriptional activation of CHO cell genes that are also upregulated in the Tras high producer clones (Table 1). We, therefore, used the Harmonizome web portal to identify potential Foxa1 target genes (Rouillard et al., 2016). Accordingly, 11 of the 25 protein‐encoding genes identified to be upregulated in Tras high producer clones were predicted to be Foxa1 target genes, including the Foxa1 gene itself (Table 1). We, therefore, tested whether these genes were upregulated upon Foxa1 overexpression in the Tras‐producing clone, revealing that Ca3 was highly upregulated upon Foxa1 overexpression at Day 8 of the fed batch. An average 98‐fold increase in Rassf9 expression was also observed, even though large variations were observed between the experiments, whereas the expression of other Foxa1 potential target genes was not significantly altered (Figure 3d). Furthermore, Erp27 was not upregulated in Foxa1‐overexpressing cells, whereas we observed an upregulation of the Tagap candidate gene (see also the accompanying paper by Pourcel, Buron, Arib, et al., 2020). Moreover, Tagap mRNA expression was increased in the Tras clone compared to the parental CHO cells at Days 0 and 8 of fed‐batch cultures, with an upregulation of 10.2‐ and 5.5‐fold, respectively (Figure S3a). Notably, we also observed Rassf9, Ca3, and Tagap mRNA upregulation in Foxa1‐overexpressing cells at Day 0 of the fed‐batch cultures, indicating that their upregulation was not a consequence of the high cell growth observed at Day 8 in Foxa1‐overexpressing cells (Figure 3e). As Ca3 has been shown to protect cells from oxidative stress (reviewed in Di Fiore, Monti, Scaloni, De Simone & Monti, 2018), we evaluated the levels of intracellular ROS using the reactive fluorescent dye carboxy‐H_2_DCFDA. Interestingly, whereas at Day 3, there was a slight increase in ROS levels in Foxa1‐overexpressing cells, ROS levels were reduced in Foxa1‐overexpressing cells at Days 6, 8, and 9 (Figure 3f).

### 3.5 Ca3 and Tagap overexpression increase Tras production

**Figure 4:**
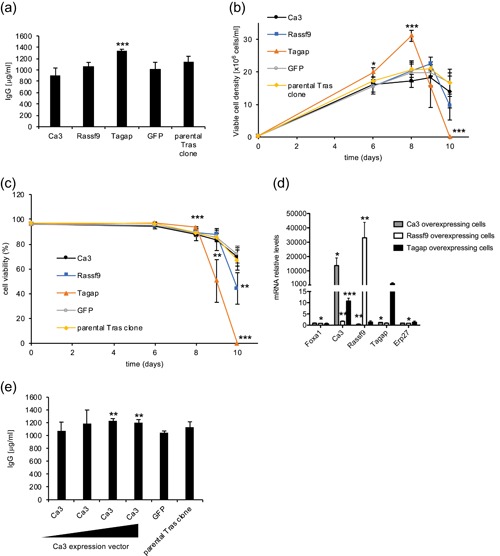
Effect of Ca3, Rassf9, and Tagap overexpression on trastuzumab (Tras) production. The parental Tras clone was stably transfected with the Ca3, Rassf9, Tagap, or GFP expression vector. (a) The Tras titers of the resulting polyclonal populations were determined after 10 days of fed‐batch cultures. Viable cell density (b) and cell viability (c) were evaluated throughout fed‐batch cultures. Error bars are shown as *SD*. n ≥ 3 for Panels a–c. (d) Quantification of the mRNA levels of candidate genes by RT‐qPCR analyses in Ca3‐, Rassf9‐ or Tagap‐expressing stable polyclonal populations. Data are presented relative to the mRNA levels in control GFP‐expressing cells. Error bars are shown as *SD*, *n* = 3. (e) The Tras clone was stably transfected with various amounts of the Ca3 expression vector (1,800, 600, 200, and 66 ng) together with an empty vector to keep the total amount of plasmid constant. The Tras titers obtained from these polyclonal populations were assessed at the end of fed‐batch cultures. Error bars are shown as *SD*, *n* = 3. mRNA, messenger RNA; qRT‐PCR, quantitative reverse transcription‐polymerase chain reaction; *SD*, standard deviation

We next hypothesized that the Tras titer increase resulting from Foxa1 overexpression could be the consequence of Ca3, Rassf9, and/or Tagap upregulation. This was tested by stably overexpressing the three candidate genes in the Tras‐producing clone and assessing the Tras titers obtained from fed‐batch cultures. Consistently, a higher Tras titer was obtained upon Tagap overexpression in the Tras‐producing clone, whereas no effect was detected from the overexpression of Ca3 or Rassf9 (Figure 4a). An increased VCD was observed at Days 6 and 8 of culture upon Tagap overexpression, with a maximum VCD of 31 million cells per ml at Day 8 (Figure 4b). However, cell viability strongly decreased starting from Day 9, and therefore there was no increase in the time integral of the VCD upon Tagap overexpression (Figure 4c and data not shown). The increase in Tras production upon Tagap overexpression was similar to the levels obtained upon Foxa1 overexpression, with a titer of 1,331 μg/ml, despite the lower cell viability upon prolonged fed‐batch cultures observed from Tagap overexpression (Figures 3a–c and 4a–c). A 28% increase in the specific productivity was observed upon Tagap overexpression (13.3 pg·cell^−1^·day^−1^ vs. 10.4 pg·cell^−1^·day^−1^; *p* = .002). A slight increase in the mRNA levels of Tras HC and LC (1.6 and 1.3, respectively) was also observed upon Tagap overexpression (Figure S4a).

Interestingly, an 11‐fold increase in Ca3 expression was also observed upon Tagap overexpression (Figure 4d). To assess if the lack of effect of Ca3 overexpression alone may result from unfavorable expression levels (Figure 4a–c), we titrated the amount of the expression vector used to establish the stable cell pools. A slight increase in the Tras titer was obtained upon the higher Ca3 overexpression levels, whereas VCD and viability were not affected (Figures 4e and S4b and data not shown). In conclusion, whereas Tagap overexpression could recapitulate the Foxa1‐mediated increase in Tras titer and temporarily improve VCD, it rather decreased cell viability at the end of fed‐batch cultures. Overall, we therefore concluded that the positive effects of Foxa1 on cell viability and cell growth in culture, and on protein titer, may result from its effect on the expression of several target genes.

### 3.6 Foxa1 also improves the production of difficult‐to‐express therapeutic proteins

**Figure 5:**
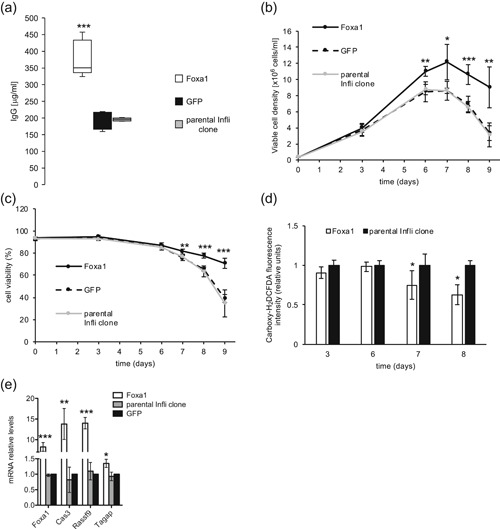
Effect of Foxa1 overexpression on infliximab production. (a) The parental infliximab clone was stably transfected with the Foxa1 or GFP expression vector, and the infliximab titers of the resulting polyclonal populations were determined after 9 days of fed‐batch cultures. Viable cell density (b) and cell viability (c) were evaluated throughout fed‐batch cultures. *n* = 5 for Panels a–c. Titers are depicted as described for Figure 2 (Panel a), whereas error bars are shown as *SD* (Panels b and c). (d) Evaluation of intracellular ROS levels using carboxy‐H_2_DCFDA for Foxa1‐overexpressing cells and for the parental infliximab‐producing clone at Days 3, 6, 7, and 8 of the fed‐batch cultures. Error bars are shown as *SD*, *n* = 3. (e) RT‐qPCR quantification of Foxa1, Ca3, Rassf9, and Tagap mRNA levels in Foxa1‐overexpressing cells, GFP‐expressing cells or in the parental infliximab clone at Day 6 of the fed batch. Error bars are shown as *SD*, *n* = 3. mRNA, messenger RNA; qRT‐PCR, quantitative reverse transcription‐polymerase chain reaction; *SD*, standard deviation

**Figure 6:**
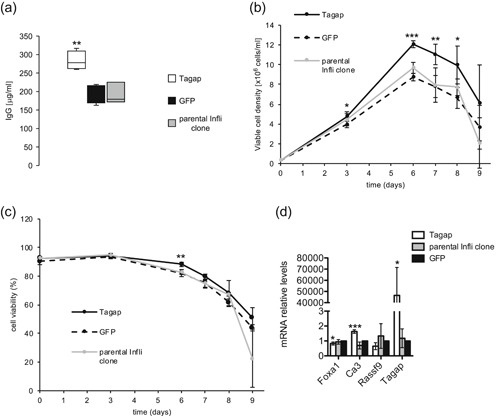
Effect of Tagap overexpression on infliximab production. (a) The parental infliximab clone was stably transfected with the Tagap or GFP expression vector, and the infliximab titers of the resulting polyclonal populations were determined after 9 days of fed‐batch cultures. Viable cell density (b) and cell viability (c) were evaluated throughout fed‐batch cultures. *n* = 4 for Panels a–c. Titers are illustrated as described in Figure 2. Error bars are shown as *SD* for Panels b and c. (d) RT‐qPCR quantification of Foxa1, Ca3, Rassf9, and Tagap mRNA levels in Tagap‐overexpressing cells, GFP‐expressing cells or in the parental clone at Day 6 of the fed batch by RT‐qPCR. Error bars are shown as *SD*, *n* = 3. mRNA, messenger RNA; qRT‐PCR, quantitative reverse transcription‐polymerase chain reaction; *SD*, standard deviation

We further assessed the effect of Foxa1 overexpression on the secretion of the difficult‐to‐express infliximab. Impressively, infliximab production was nearly doubled upon Foxa1 overexpression, where an average titer of 378 μg/ml was obtained, upon an 8.2‐fold increase of Foxa1 mRNA levels (Figures 5a and 5e). Notably, Foxa1‐overexpressing cells showed a significantly increased VCD starting from Day 6 until Day 9, reaching a maximum VCD of 12.2 million cells/ml at Day 7, whereas control cells only reached a VCD of 8.6 million cells/ml (Figure 5b). Consistently, we observed that cell viability remained significantly higher in Foxa1 expressing cells, preventing the crash of cell viability observed from Day 7 with the control cells (Figure 5c). This was accompanied by a decrease in the accumulation of ROS at Days 7 and 8 in the Foxa1‐overexpressing cells (Figure 5d). Notably, a 38% fold increase in the specific productivity was observed upon Foxa1 overexpression (6.6 pg·cell^−1^·day^−1^ vs. 4.8 pg·cell^−1^·day^−1^; *p* = .028), indicating that the Foxa1‐mediated increase in titer was due to both an increased time integral of the VCD and increased specific productivity. Similar to what was observed upon Foxa1 overexpression in the Tras‐producing clone, Ca3, Rassf9, and Tagap mRNA levels were also upregulated upon Foxa1 overexpression (Figure 5e). Consistently, we obtained a 45% increase in infliximab production upon Tagap overexpression, yielding an average titer of 283 μg/ml (Figure 6a). Therefore, although Tagap overexpression could recapitulate the Foxa1‐mediated increase of the Tras titer, it only partially mimicked the Foxa1‐induced infliximab titer increase. As observed for the Tras‐producing clone, Tagap overexpression resulted in a rapid increase in VCD for the infliximab clone, with a maximum VCD of 12 million cells/ml at Day 6 (Figure 6b). However, in contrast to Foxa1‐overexpressing cells, cell viability remained mostly unchanged upon Tagap overexpression (Figure 6c). Moreover, there was no increase in the specific productivity upon Tagap overexpression (4.8 pg·cell^−1^·day^−1^ vs. 4.3 pg·cell^−1^·day^−1^; *p* = .19), indicating that, in this case, the Tagap‐mediated increase in titer was solely due to the increased time integral of the VCD. Notably, Tagap overexpression in the infliximab‐producing clone also yielded an upregulation of Ca3 mRNA levels (Figure 6d). Taken together, these results indicated that Foxa1 overexpression can be used to increase the production levels of difficult‐ as well as easy‐to‐express therapeutic proteins in CHO cells, and that this effect may result in part from the Foxa1‐mediated increase in Tagap expression levels.

## 4 DISCUSSION

In this study, we identified the ER‐located protein Erp27 as being involved in the high‐level production of both easy‐to‐express and difficult‐to‐express therapeutic proteins. Despite the fact that Erp27 is a redox‐inactive member of the PDI family, it is likely to participate in protein folding, as it selectively binds to unfolded proteins and interacts with the disulfide isomerase Erp57 (Alanen et al., 2006; Kober et al., 2013). Notably, difficult‐to‐express proteins are prone to misfolding, and the unfolded protein response (UPR) was shown to be activated upon expression of difficult‐to‐express proteins (reviewed in Hansen et al., 2017). Thus, Erp27 and Erp57 overexpression likely contribute directly to decrease the accumulation of misfolded difficult‐to‐express proteins, thereby preventing or delaying UPR‐induced apoptosis. This well explains the increase in cell viability and VCD upon Erp27 and Erp57 co‐overexpression in cells expressing difficult‐to‐express proteins. Although Erp27 and Erp57 were shown to be upregulated upon ER stress (Bargsted, Hetz, & Matus, 2016; Kober et al., 2013), this upregulation might not be sufficient to deal with the large quantity of misfolded recombinant proteins. Besides increasing the yield of therapeutic proteins, overexpression of Erp27 and Erp57 might also prevent quality issues of the product, as the antibody quality was found to decrease together with cell viability (Kaneko, Sato, & Aoyagi, 2010). In contrast, the high production of the Tras antibody did not trigger a full UPR response (Le Fourn et al., 2014), which is consistent with the finding that Erp27 overexpression, combined or not with Erp57, did not improve cell viability and had no or little effect on VCD in these conditions. Nevertheless, the folding capacity of CHO cells might still represent a bottleneck in these conditions, as indicated by the fact that Erp27 moderate overexpression increased the Tras titer.

Although protein folding in the ER was demonstrated to be a limiting step for the production of several therapeutic proteins, conflicting results were published concerning the effect of PDI and Erp57 overexpression on therapeutic protein production (reviewed in Hansen et al., 2017). It will be, therefore, interesting to characterize the contribution of the Erp27–Erp57 complex to therapeutic protein folding with regard to PDI and Erp57‐CNX/CLRT, considering that the same domain of Erp57 is used for binding to CRT/CNX and to Erp27 (Alanen et al., 2006).

We also identified the pioneering transcription factor Foxa1 as a candidate gene of interest for cell metabolism engineering, as it increases cell viability, VCD, specific productivity, and the production of both easy‐to‐express and difficult‐to‐express therapeutic proteins when overexpressed. This effect may result in part from the Foxa1‐mediated Tagap upregulation. Indeed, when overexpressed, Tagap could increase the specific productivity of easy‐to‐express therapeutic proteins and the time IVCD of difficult‐to express therapeutic proteins. Consequently, an increase in the titer of easy‐to‐express and difficult‐to‐express therapeutic proteins was observed. Tagap is a signaling protein member of the Rho GTPase‐activating protein (GAP) family. In thymocytes, it was shown to regulate the abundance of active RhoA, thus promoting cytoskeleton reorganization and release of β1‐integrin‐mediated adhesion allowing thymocytes migration from the cortex to the medulla (Duke‐Cohan et al., 2018). Moreover, Tagap and the cardiac muscle actin alpha (ACTC1) were found to be upregulated in vitamin B5‐selected cells producing therapeutic proteins at very high levels, and Tagap overexpression was shown to increase the expression of ACTC1, which in turn increases the production of various therapeutic proteins (see accompanying paper, Pourcel, Buron, Arib, et al., 2020). Interestingly, spherical integrin clustering, as well as an increase in actin content and formation of a spherical actin sheath, was observed in suspension‐adapted CHO cells (Walther, Whitfield, & James, 2016). Increased expression of Tagap could, therefore, contribute to improve the actin‐mediated adaptation of cells in a suspension environment. Tagap upregulation could also contribute to improve therapeutic protein secretion as the actin cytoskeleton is involved in the regulation of the secretory pathway (Stamnes, 2002). Notably, another candidate gene upregulated in Tras high producer clone, Arhgap42, is a Rho GAP that was shown to localize to actin stress fibers and focal adhesions and to promote cell motility (Hu et al., 2018; Luo et al., 2017). Furthermore, Arhgap42 is also a Foxa1 target gene. Thus, it will be interesting to test if increased Arhgap42 expression may also increase titer and VCD.

Ca3 upregulation was also observed in the easy‐ and difficult‐to‐express protein high producer clones as well as in Foxa1‐overexpressing cells and to a lesser extent in Tagap‐overexpressing cells. Notably, Ca3 was shown to inhibit H_2_O_2_‐induced apoptosis and to reduce H_2_O_2_‐induced ROS activity (Raisanen et al., 1999; Shi et al., 2018). It was also shown to protect cells against hypoxic stress (reviewed in Di Fiore et al., 2018). Importantly, the accumulation of ROS was observed during fed‐batch cultures, and oxidative stress was shown to affect the yield and galactosylation of antibodies (Ha, Hansen, Kol, Kildegaard, & Lee, 2018). Moreover, the addition of the antioxidants baicalein or S‐sulfocysteine in fed‐batch cultures improved cell viability and antibody production in fed‐batch cultures (Ha et al., 2018; Hecklau et al., 2016). Consistently, we found a decrease in ROS accumulation in Foxa1‐overexpressing cells during the last days of the fed‐batch cultures and an increase in cell viability. In contrast, although Ca3 overexpression resulted in an increased Tras titer, we did not observe any positive effect on cell viability. A possible explanation is that Ca3 was not overexpressed at the correct level. It is also possible that the Foxa1‐mediated increase in cell viability requires the activation of other genes. A possible candidate is CDK15, which is also upregulated in the Tras high producer clones and was shown to protect cells against apoptosis (Park, Kim, Kim, & Chung, 2014); however, it remains to be tested whether CDK15 is a Foxa1 target gene.

Finally, Rassf9 upregulation was also observed in the easy‐ and difficult‐to‐express high producer clones as well as in Tras‐ and infliximab‐producing cells upon Foxa1 overexpression. Rassf9 was shown to associate with recycling endosomes and was proposed to regulate vesicular trafficking via its interaction with integral membrane proteins (Chen, Johnson, & Milgram, 1998). Although its overexpression did not result in an increase in therapeutic protein titer, it is, however, possible that it is involved in the secretion of therapeutic proteins.

Overall, we observed that the upregulation of several CHO cell genes contributes in improving the production yields of various easy‐ and difficult‐to‐express therapeutic proteins. Interestingly, several of these CHO genes appear to be upregulated by the Foxa1 transcriptional activator. We, therefore, conclude that Foxa1 increased expression may elicit a transcriptional program that is favorable for high‐level therapeutic protein production, and this provides a convenient approach to improve the production of recombinant proteins of interest.

## CONFLICT OF INTERESTS

V. L., A. R., P. A. G. and I. B. are employed by—and N. M. is a consultant of—Selexis SA, a company that generates Chinese hamster ovary cell clones expressing therapeutic proteins. Selexis SA did not contribute to or influence the writing of the manuscript.

## AUTHOR CONTRIBUTIONS

A. B. designed the project; A. B., V. L., J. M., A. R., and I. B. performed the experiments; A. B., P. A. G. and N. M. analyzed the results and A. B. and N. M. wrote the paper.

## Supporting information

Supporting information
