# Supplementary material for: Overexpression of transcription factor Foxa1 and target genes remediate therapeutic protein production bottlenecks in Chinese hamster ovary cells
Source: Biotechnol Bioeng. 2020 Feb 23;117(4):1101–16. doi: 10.1002/bit.27274 (PMC7079004; doi:10.1002/bit.27274)
Supplement: Supplementary file 1 — Supporting information [file BIT-117-1101-s001.pdf]

# Supplementary Data

The supplementary data includes:

Supplementary Materials and Methods

Supplementary reference

Supplementary Figures S1-S4

Supplementary Table S1

## Supplementary Materials and Methods

### Fed batch cultures

Cells were seeded at 300'000 cells/ml in 50-ml Tubespin bioreactor (TPP, Switzerland) containing 5ml of culture medium supplemented with 16% of HyClone Cell Boost 5 supplement (GE healthcare). Cells were fed at day 2, 3, 6, 7 and 8 with 16% of HyClone Cell Boost 5 supplement. Viable cell density was evaluated using the LUNA Automated Cell Counter (Logos biosystems) after staining of the cells with 0.1% of Erythrosin B. IgG titers were measured in cell culture supernatants by sandwich ELISA.

### ELISA

Antibody concentrations in cell culture supernatants were measured by sandwich ELISA. Briefly the 96 well ELISA plate (Nunc Maxisorp, Invitrogen) was coated with the goat anti-human kappa light chain antibody (1:500 dilution in PBS; Bethyl Laboratories Inc.) and incubated overnight at 4°C. Then, after 3 washes, the samples were loaded onto the plate in 1% casein hydrolysate containing blocking solution and incubated for 30 minutes at 37°C. The plate was washed 3 times before addition of the Human IgG-Fc fragment antibody alkaline phosphatase conjugated (1:1000 dilution in blocking solution; Bethyl Laboratories Inc.) and incubated for 30 minutes at 37°C. Next, the plate was washed 4 times before the addition of a p-Nitrophenyl Phosphate substrate solution (Sigma-Aldrich) and incubated in the dark for 15 minutes at room temperature. The reaction was stopped by the addition of 3M NaOH. Absorbance was read in a microplate reader (Spectra Max 340, Molecular Devices) at wavelengths of 405/490 nm. Analyzes of etanercept concentration in cell culture supernatants were performed by sandwich ELISA with the following changes: the plate was coated with goat anti-human IgG (Fc specific), F(ab’)2 fragment antibody (dilution 1:1000;

Sigma-Aldrich) and incubated overnight at 4°C. The plate was incubated in blocking solution for 1h at room temperature followed by incubation with the samples for 1h at room temperature. The protein A–peroxidase (dilution 1:10’000; Sigma-Aldrich) was then added and incubated for 1h at room temperature. The SureBlue Reserve™ Microwell Substrate (KPL Inc.) was added for 30 minutes and the reaction was stopped by addition of 1N HCl. Absorbance was read in the ELISA reader (Spectra Max 340, Molecular Devices) at wavelength of 450 nm.

**Real-time quantitative RT-PCR**

Total RNA was extracted using the NucleoSpin™ RNA kit (Macherey-Nagel) and reverse transcribed into cDNA using the GoScript Reverse transcription System (Promega) according to the manufacturer's instructions. Real-time qPCR was performed using the LightCycler® 480 SYBR Green I Master and the LightCycler 480 II instrument (Roche). Transcript levels were normalized to SDHA mRNA levels. Primers are listed in Table S1.

**Foxa1 target gene analysis**

The Harmonizome web portal was used to identify potential Foxa1 target genes (Rouillard et al., 2016). Genes were considered to be Foxa1 target genes according to ChIP-seq datasets (ENCODE Transcription Factor Targets dataset) and to low or high-throughput transcription factor functional studies (TRANSFAC Curated Transcription Factor Targets Dataset).

**Statistical methods**

Unpaired two-tailed Student’s t-test was used for statistical analyses. A single asterisk indicates a p-value ≤ 0.05, two asterisks indicate a p-value ≤ 0.01, three asterisks indicate a p-

value ≤ 0.001. Unless specified otherwise, the number of biological replicates are indicated in the figure legends as the n numbers.

## Supplementary Reference

Rouillard, A.D., Gundersen, G.W., Fernandez, N.F., Wang, Z., Monteiro, C.D., McDermott, M.G., and Ma'ayan, A. (2016). The harmonizome: a collection of processed datasets gathered to serve and mine knowledge about genes and proteins. Database (Oxford) *2016*.

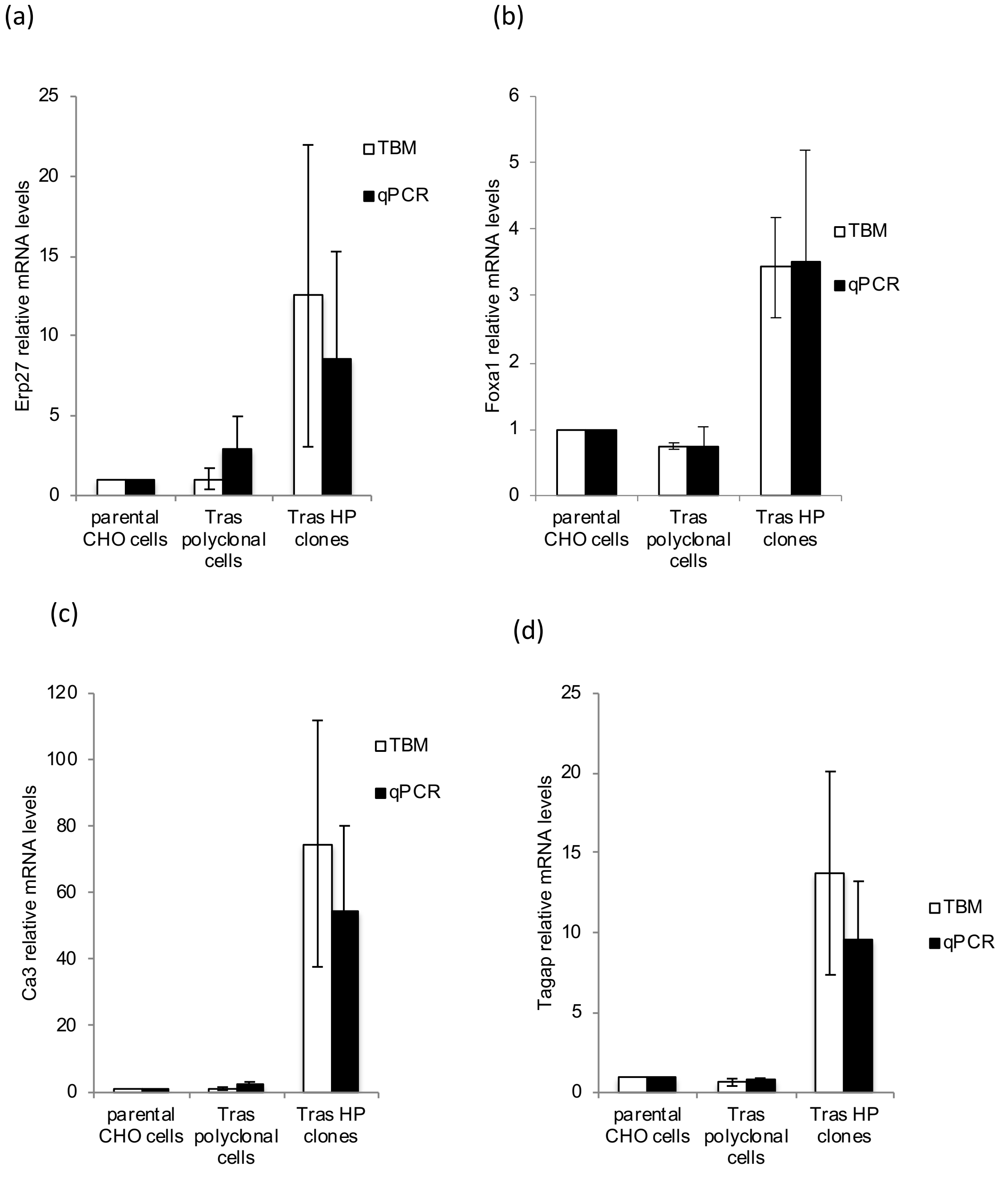


**Supplementary Figure 1. mRNA levels of candidate genes obtained from the RNASeq analysis or using RT-qPCR analysis.** mRNA levels of Erp27 (**a**), Foxa1 (**b**), Ca3 (**c**) and Tagap (**d**) in parental CHO cells, Tras polyclonal cells and Tras high producer (HP) clones analyzed by RNASeq and shown in transcripts per kilobase million (TBM) or analyzed using RT-qPCR. Data are presented relative to parental CHO cells. Error bars are shown as SD. Three biological replicates were used for the Tras high producer clones, while three technical replicates were used for parental CHO cells and for the Tras-producing polyclonal cell population.

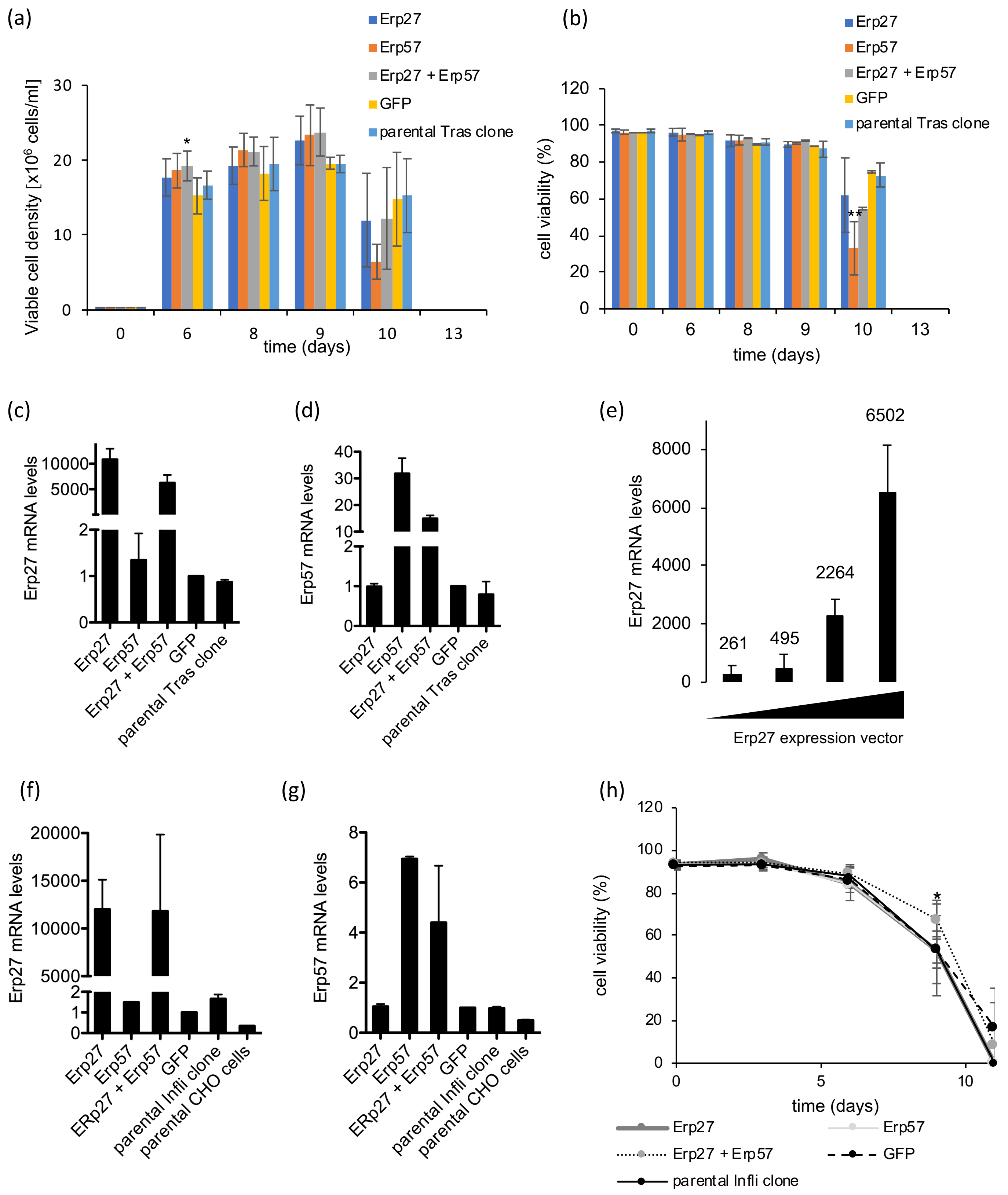


**Supplementary Figure 2. Fed-batch culture analyses and mRNA levels of cells producing easy-to-express or difficult-to-express therapeutic proteins and overexpressing Erp27, Erp57 or both.** Viable cell density (**a**) and cell viability (**b**) of the trastuzumab producing clone stably transfected with the expression vectors for Erp27 and/or Erp57 during fed-batch cultures. Error bars are shown as SD, n=3. Quantification of Erp27 (**c**) and Erp57 (**d**) mRNA levels in the different cell populations by qRT-PCR. Data are presented relative to the mRNA levels of control GFP-expressing cells. Error bars are shown as SD, n=3. (**e**) Quantification by qRT-PCR of Erp27 mRNA levels in the Tras clone stably transfected with decreasing amounts of Erp27 expression vector and with an empty vector to keep the total amount of plasmid constant. Data are presented relative to Erp27 mRNA levels in control cells. Error bars are shown as SD, n=2. (**f and g**) qRT-PCR quantification of Erp27 and Erp57 mRNA levels in the infliximab-producing clone stably transfected with expression vectors for Erp27 and Erp57. Data are presented relative to mRNA levels in control cells. Error bars are shown as SD, n=2. (**h**) Cell viability of the different cell populations analyzed during fed-batch. Error bars are shown as SD, n≥4.

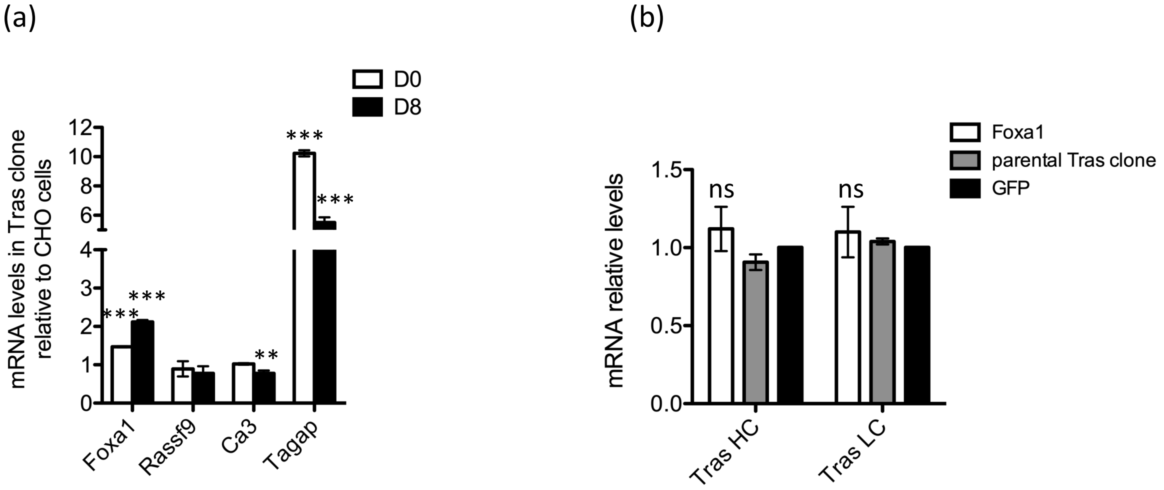


**Supplementary Figure 3. mRNA levels of candidate genes and trastuzumab heavy chain (HC) and light chain (LC) transgenes during fed-batch cultures.** (**a**) RT-qPCR quantification of Foxa1, Rassf9, Ca3 and Tagap mRNA levels at day 0 and day 8 of fed-batch cultures in the trastuzumab producing clone. Data are presented relative to the mRNA levels in CHO cells. Error bars are shown as SD, n=3. (**b**) RT-qPCR quantification of Tras HC and LC mRNA levels in Foxa1 overexpressing cells, GFP expressing cells or in the parental Tras clone at day 8 of fed-batch cultures. Data are presented relative to the mRNA levels in control GFP-expressing cells. Error bars are shown as SD, n=3.

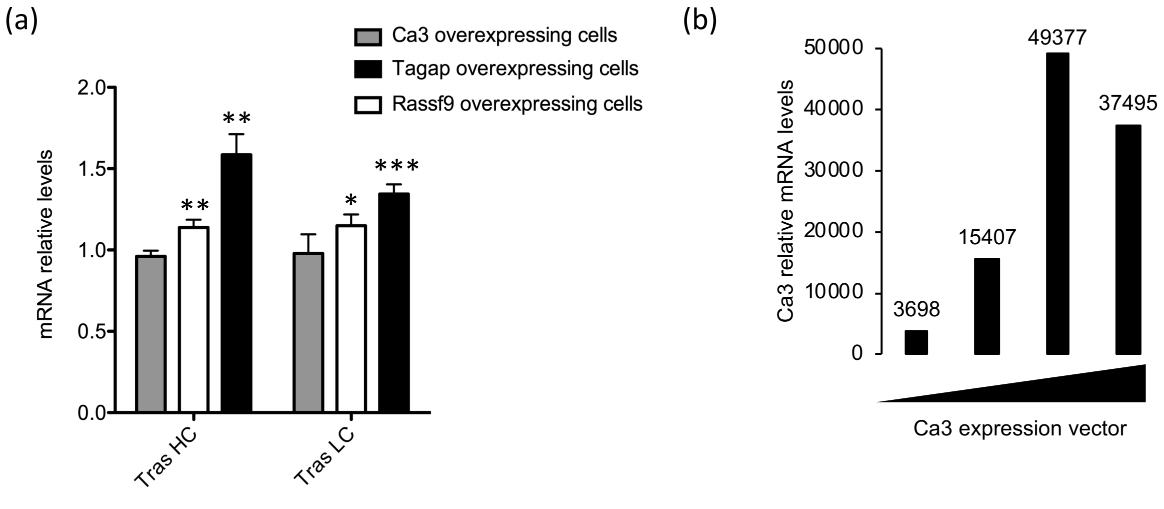


**Supplementary Figure 4. Analyses of trastuzumab heavy chain (HC) and light chain (LC) transgene and Ca3 mRNA levels.** (**a**) RT-qPCR quantification of Tras immunoglobulin heavy and light chain mRNA levels in Cas3, Rassf9 and Tagap-overexpressing cells. Data are presented relative to Tras heavy chain and light chain mRNA levels in control GFP-expressing cells. Error bars are shown as SD, n=3. (**b**) RT-qPCR quantification of Ca3 mRNA levels in the Tras clone stably transfected with various amounts of Ca3 expression vector and with an empty vector to keep the total amount of plasmid constant. Data are presented relative to Ca3 mRNA levels in control GFP-expressing cells.

| **Supplementary Table S1**. List of primers used for RT-qPCR analysis | | |
|---|---|---|
| Gene | Forward primer | Reverse primer |
| Erp27 | TGCAGCTGGCTTATTTAACACC | CCTGGAAGAGCTTAGCTGCC |
| Erp57 | TGGAACTCACGGACGAAAACT | AGGGGCGAAGAACTCGACTA |
| SDHA | TGGCGTGGATGTCACTAAGG | CAGCACCTGCCCTTTGTAGT |
| Foxa1 | AAAGGGGACCCCCACTACTC | TGCCTTGAAGTCCAGCTTGT |
| Ca3 | GGAATCGCTGTTGTTGGCAT | GAGCCTCCTTGCCCTTAGTC |
| Rassf9 | TGGCACAGCTAGAAGAACGG | TCTTCACTTCCGTCGATGCC |
| Epcam | TGTTTGGTGATGAAGGCGGA | TCGTTGTTCTGTATGGCCCC |
| Pcdhb3 | CTGGGTCTAGGCGCTATTCTG | CTACCCTGAGCCCCAAATCC |
| Slc25a23 | TCCGAGATTCAGCAGAGCTTC | CGCCATTCCTGCCAATCAATG |
| Pde1a | TGGGTGTTTCTTGGGGTAGG | ACCCACCAAGAGTCACGTTG |
| Edn1 | AGAAGGTTGGAGGCCATCAC | TGCTCGGTTGTGTGTCAACT |
| Fras1 | CACACCCACCTGGAAAGTCA | GTTAGGCCATCTTCCCGAGC |
| Frk | GCCCAGTCCCCTCTTGATTT | GCAGAGCTGAGAGAGTTCCC |
| Arhgap42 | CAGTTCAACTTGCAGAATACAAGG | TGGCTGGGTGGTCTGTAATC |
| Tagap | GCCCACCATCCTACGAAGAG | GAGCCGTGTTCCATTTGAGC |
| Tras HC | GACTCCGATGGGTCGTTCTT | CATGACGGAGCAGGAGAACA |
| Tras LC | GCGGACTACGAGAAGCACAA | CGGTTGAACGACTTGGTCAC |
